# ADVANCING AGING EQUITY AMONG OLDER BOSTONIANS

**DOI:** 10.1093/geroni/igad104.1511

**Published:** 2023-12-21

**Authors:** Adriana Hernandez, Caitlin Coyle

**Affiliations:** University of Massachusetts Boston, Boston, Massachusetts, United States; University of Massachusetts Boston, Boston, Massachusetts, United States

## Abstract

More than five years after the City of Boston started its journey towards becoming an Age and Dementia Friendly community, the initiative and its collaborators have been working to ensure that the initiative and its many contributions are equally realized by all Bostonians. Drawing on the concept of aging equity--that all older adults, despite their life course histories, have equitable access to programs and services that help them age well. To achieve this means removing obstacles to accessing community features that support healthy aging, establishing social and civic engagement opportunities, ensuring safe environments, establishing access to healthcare, and disseminating knowledge of available supports and services. This presentation will describe the research and community engagement that led to the development of an aging equity conceptual framework and examples of how it is being operationalized in the City of Boston. For example, we will share the ways in which outreach and communication strategies have been altered to more equitable practices including efforts to build trust, engage in cultural translation of programs and services, and consider new channels of information sharing.

